# α-synuclein and tau: interactions, cross-seeding, and the redefinition of synucleinopathies as complex proteinopathies

**DOI:** 10.3389/fnins.2025.1570553

**Published:** 2025-03-27

**Authors:** Francisco J. Padilla-Godínez, Eunice Ruth Vázquez-García, María Isabel Trujillo-Villagrán, Luis O. Soto-Rojas, Marcela Palomero-Rivero, Omar Hernández-González, Francisco Pérez-Eugenio, Omar Collazo-Navarrete, Oscar Arias-Carrión, Magdalena Guerra-Crespo

**Affiliations:** ^1^Laboratory of Regenerative Medicine, Department of Physiology, Faculty of Medicine, National Autonomous University of Mexico, Mexico City, Mexico; ^2^Department of Mathematics and Physics, Western Institute of Technology and Higher Education, San Pedro Tlaquepaque, Jalisco, Mexico; ^3^Laboratory of Molecular Pathogenesis, Laboratory 4, Building A4, Medical Surgeon Career, Faculty of Higher Studies Iztacala, National Autonomous University of Mexico, Mexico City, Mexico; ^4^Department of Neurodevelopment and Physiology, Institute of Cell Physiology, National Autonomous University of Mexico, Mexico City, Mexico; ^5^Computing Unit, Institute of Cell Physiology, National Autonomous University of Mexico, Mexico City, Mexico; ^6^National Laboratory of Genomic Resources, Institute of Biomedical Research, National Autonomous University of Mexico, Mexico City, Mexico; ^7^Movement and Sleep Disorders Unit, Dr. Manuel Gea González General Hospital, Mexico City, Mexico

**Keywords:** α-synuclein, tau, synucleinopathy, tauopathy, proteinopathy, Lewy bodies

## Abstract

Neurodegenerative diseases are characterized by protein aggregation and overlapping pathologies, challenging traditional classifications and highlighting shared underlying mechanisms. Parkinson’s disease and related synucleinopathies, including Lewy body dementia and multiple system atrophy, highlight the interplay between α-synuclein and tau, two key proteins implicated in these disorders. Recent studies reveal that tau and α-synuclein co-aggregate, interact synergistically, and propagate via prion-like mechanisms, exacerbating neuronal dysfunction. This review examines the physiological roles and pathological transitions of tau and α-synuclein, emphasizing their roles in microtubule dynamics, synaptic regulation, and the structural heterogeneity of aggregates. Evidence from post-mortem brains, transgenic models, and proteomic analyses underscores the significance of soluble oligomers as primary neurotoxic species and explores the diverse molecular composition of Lewy bodies and glial cytoplasmic inclusions. The co-localization of tau and α-synuclein, influenced by genetic factors and post-translational modifications, offers insights into shared mechanisms across synucleinopathies and tauopathies. These findings advocate for integrated therapeutic strategies targeting protein cross-seeding and proteostatic disruption while preserving physiological roles. By framing neurodegeneration as a collapse of proteostatic networks rather than isolated proteinopathies, this work proposes a paradigm shift toward understanding and treating complex neurodegenerative disorders.

## Highlights

- Misfolded tau is a key feature in synucleinopathies, including Parkinson’s disease, Lewy body dementia, and multiple system atrophy.
- The interaction between α-synuclein and tau occurs physiologically and pathologically, driving neurodegenerative processes.
- α-Synuclein inclusions in synucleinopathies contain diverse co-aggregating proteins beyond α-synuclein itself.
- The study of co-aggregating proteins offers valuable insights into the mechanisms underlying neurodegenerative diseases.
- Shifting the research paradigm from isolated proteinopathies to exploring molecular interactions can reveal more appropriate disease classifications, patient stratifications and, ultimately, therapeutic targets.

## 1 Introduction

With rising global life expectancy, the prevalence of neurodegenerative diseases has increased significantly, placing a considerable burden on healthcare systems worldwide (Zahra et al., 2020). These disorders are characterized by the progressive degeneration of specific neuronal populations, leading to severe functional decline and reduced quality of life for millions of individuals (Wilson et al., 2023). Shared neuropathological hallmarks, such as protein aggregates within the central nervous system (CNS), have reclassified these disorders as proteinopathies (Chen et al., 2022), that is to say, conditions where the proteostasis—the dynamic regulation of a balanced, functional proteome through biological pathways within cells that control the biogenesis, folding, trafficking, and degradation of proteins present within and outside the cell—of one or more proteins is lost (Powers et al., 2009). Despite differences in the specific proteins involved, a unifying feature is their propensity to misfold, form insoluble aggregates, and trigger cellular dysfunction through oxidative stress, mitochondrial impairment, and neuroinflammation (Dugger and Dickson, 2017). These processes contribute to progressive neurodegeneration in specific brain regions, emphasizing shared frameworks that guide integrated research efforts and therapeutic strategies (Chen et al., 2022).

Among proteinopathies, Parkinson’s disease (PD) stands as the second most common neurodegenerative disorder, affecting approximately 1% of individuals over the age of 60 (Grotewold and Albin, 2024). The etiology of PD is multifactorial, involving genetic mutations in *SNCA*, *LRRK2*, and *PARK2* genes, as well as environmental exposures such as pesticide use and traumatic brain injuries (Periñán et al., 2022). Clinically, PD is characterized by motor symptoms, including tremor, bradykinesia, and rigidity, arising primarily from dopaminergic neuronal loss in the substantia nigra (Furukawa et al., 2022). However, PD extends beyond motor dysfunction, with non-motor symptoms such as cognitive decline, autonomic dysfunction, and sleep disturbances critically shaping its clinical impact. These features overlap with a spectrum of disorders referred to as parkinsonism, which includes Parkinson’s disease dementia (PDD), Lewy body dementia (LBD), and multiple system atrophy (MSA). Other neurodegenerative diseases, such as Alzheimer’s disease (AD), also exhibit shared symptoms and pathological overlaps, with studies showing a significant proportion of PD patients developing cognitive impairments and AD patients displaying motor symptoms characteristic of parkinsonism (McKeith et al., 2004; Weisman and McKeith, 2007; Horvath et al., 2014; Foguem and Manckoundia, 2018; Aarsland et al., 2021). These connections have shifted research paradigms toward identifying shared mechanisms underpinning neurodegeneration across diseases (Chen et al., 2022).

**Figure 1:**
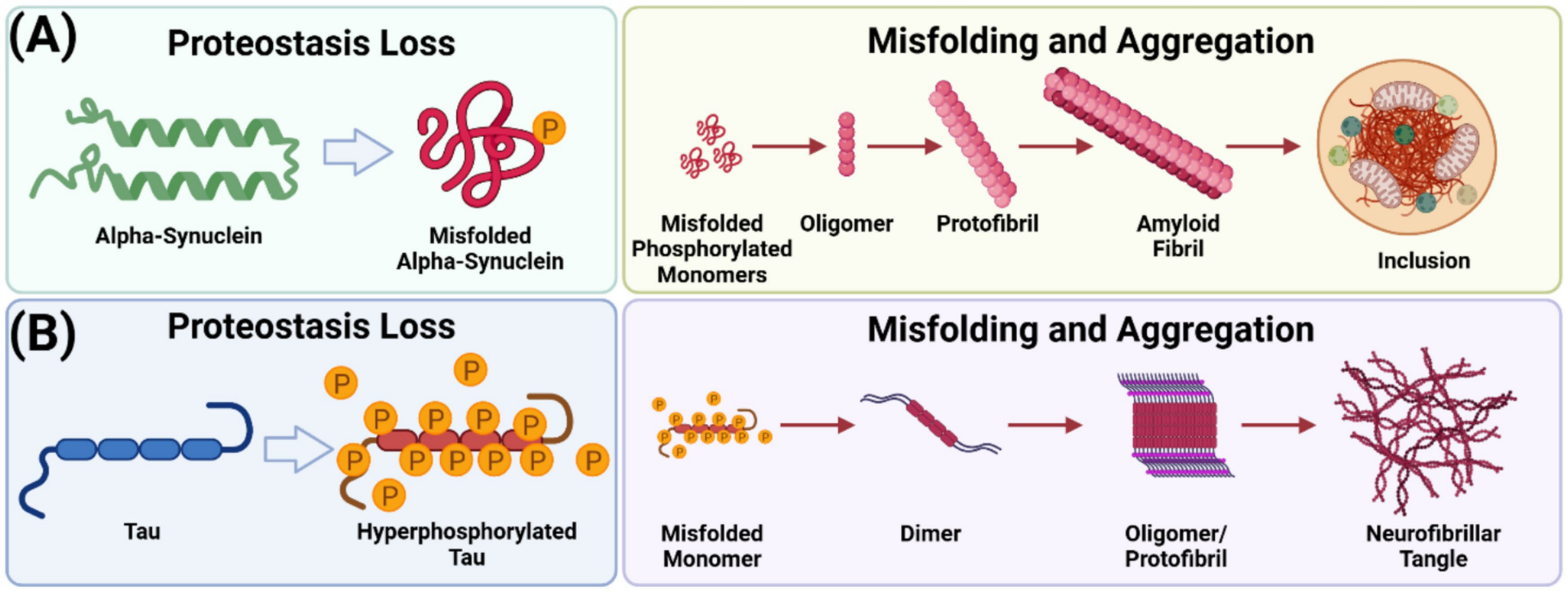
α-Synuclein and tau aggregation pathways. **(A)** Phosphorylated soluble α-syn monomers transition through intermediate, oligomeric forms before assembling into insoluble fibrillar aggregates, characterized by unique β-sheet-rich secondary structures. This process generates a structurally diverse range of aggregates and cellular inclusions. **(B)** Hyperphosphorylated tau monomers aggregate into dimers, rapidly forming oligomers and fibrillate into protofibrils. Unlike α-syn, tau aggregation does not culminate in inclusion formation—a figure created with BioRender.com.

Research on PD has increasingly focused on its classification as a proteinopathy, with α-synuclein (α-syn) aggregation playing a central role. Soluble α-syn monomers undergo pathological fibrillization into β-sheet-rich insoluble aggregates, forming structures such as Lewy bodies (LBs) (Vidović and Rikalovic, 2022). Traditionally, LBs have been suggested to disrupt synaptic function by impairing vesicle docking, fusion, and neurotransmitter release (Srinivasan et al., 2021; Menšíková et al., 2022); however, whether LBs are a pathological consequence of α-syn misfolding and aggregation, or a cytoprotective mechanisms remains a debate. Notably, LBs are not solely composed of α-syn; they include various misfolded proteins and organelles, with tau—a microtubule-stabilizing protein—playing a notable role. Hyperphosphorylated tau, a hallmark of tauopathies such as AD, aggregates through distinct stages from monomer to fibril, suggesting pathological synergy between tau and α-syn in PD (Zhang et al., 2018; Wegmann et al., 2021; Rawat et al., 2022; Hu et al., 2023; Zheng et al., 2024). While the relationship between these proteins remains unclear, evidence points to toxic interactions that exacerbate cellular dysfunction (Figure 1). Investigating the spectrum of misfolded proteins in LBs, rather than focusing solely on α-syn, offers an opportunity to uncover novel mechanisms and comprehensive therapeutic strategies (Chen et al., 2022).

This review aims to critically examine the interplay between α-syn and tau, emphasizing their shared roles in the molecular pathogenesis of PD and related disorders. By exploring the molecular heterogeneity of LBs and glial cytoplasmic inclusions (GCIs), this work identifies overlapping pathways that may serve as targets for novel interventions. Additionally, the review advocates for a shift in research perspectives, positioning neurodegenerative diseases as interconnected proteinopathies rather than isolated entities. By fostering a collaborative, integrated approach, this work seeks to accelerate the development of multifaceted treatment strategies for these complex disorders.

## 2 Synucleinopathies: the role of α-synuclein in neurodegeneration

### 2.1 α-Synuclein: physiological functions and structural dynamics

A thorough analysis of synucleinopathies must begin by considering α-syn as a pathological entity and a physiologically active protein with dynamic structural properties underpinning its diverse roles in neuronal function. While the complete physiological functions of α-syn remain under investigation, compelling evidence highlights its central role in synaptic regulation.

A primary function of α-syn is its involvement in synaptic vesicle trafficking and neurotransmitter release. Localized predominantly at presynaptic terminals, α-syn interacts with synaptic vesicles to promote their clustering. This interaction is thought to regulate vesicle availability and facilitate neurotransmitter release, thereby influencing synaptic plasticity (Sharma and Burré, 2023). Additionally, α-syn modulates the assembly of the SNARE (soluble N-ethylmaleimide-sensitive factor attachment protein receptor) complex, which is essential for synaptic vesicle exocytosis (Das et al., 2022). Stabilizing the SNARE complex, α-syn ensures efficient synaptic transmission and neuronal communication (Gao et al., 2023).

Emerging evidence further implicates α-syn in maintaining synaptic vesicle pools, lipid metabolism, and mitochondrial integrity. Its ability to bind lipids and regulate membrane curvature is critical for vesicle formation and trafficking (Kulkarni et al., 2022). Dysfunction in these roles can lead to synaptic and neuronal impairments, providing a potential link to neurodegenerative disease mechanisms (Johnson and Zeno, 2023).

### 2.2 Preservation of proteostasis in α-synuclein

The intracellular proteostasis of α-syn is regulated by inherent monitoring mechanisms, primarily involving the ubiquitin-proteasome system (UPS) and the lysosomal autophagy system (LAS). LAS appears to play a more significant role in eliminating oligomeric aggregates. A recent study found that inclusion formation is driven by α-syn concentration, while their removal occurs through the autophagy pathway, as demonstrated by quantitative analysis of α-syn inclusion formation and clearance in a yeast model of PD (Perrino et al., 2019). The accumulation of α-syn is primarily linked to the dysfunction of these degradation pathways (Xilouri et al., 2013). Additionally, abnormal proteins can directly or indirectly disrupt UPS processes, further impairing associated pathways and ultimately leading to irreversible neuronal protein imbalance and degeneration (Cookson, 2009; Sato et al., 2018).

However, the intrinsic structural flexibility of α-syn, characterized by its disordered nature, predisposes it to pathological self-aggregation. When the proteostasis mechanisms fail, α-syn aggregates into high-molecular-weight structures, which have been observed to coincide with neuronal dysfunction and degeneration. These aggregates, characterized by diverse structural variants, are central pathological agents in synucleinopathies (Bernal-Conde et al., 2020; Rouaud et al., 2021).

**Figure 2:**
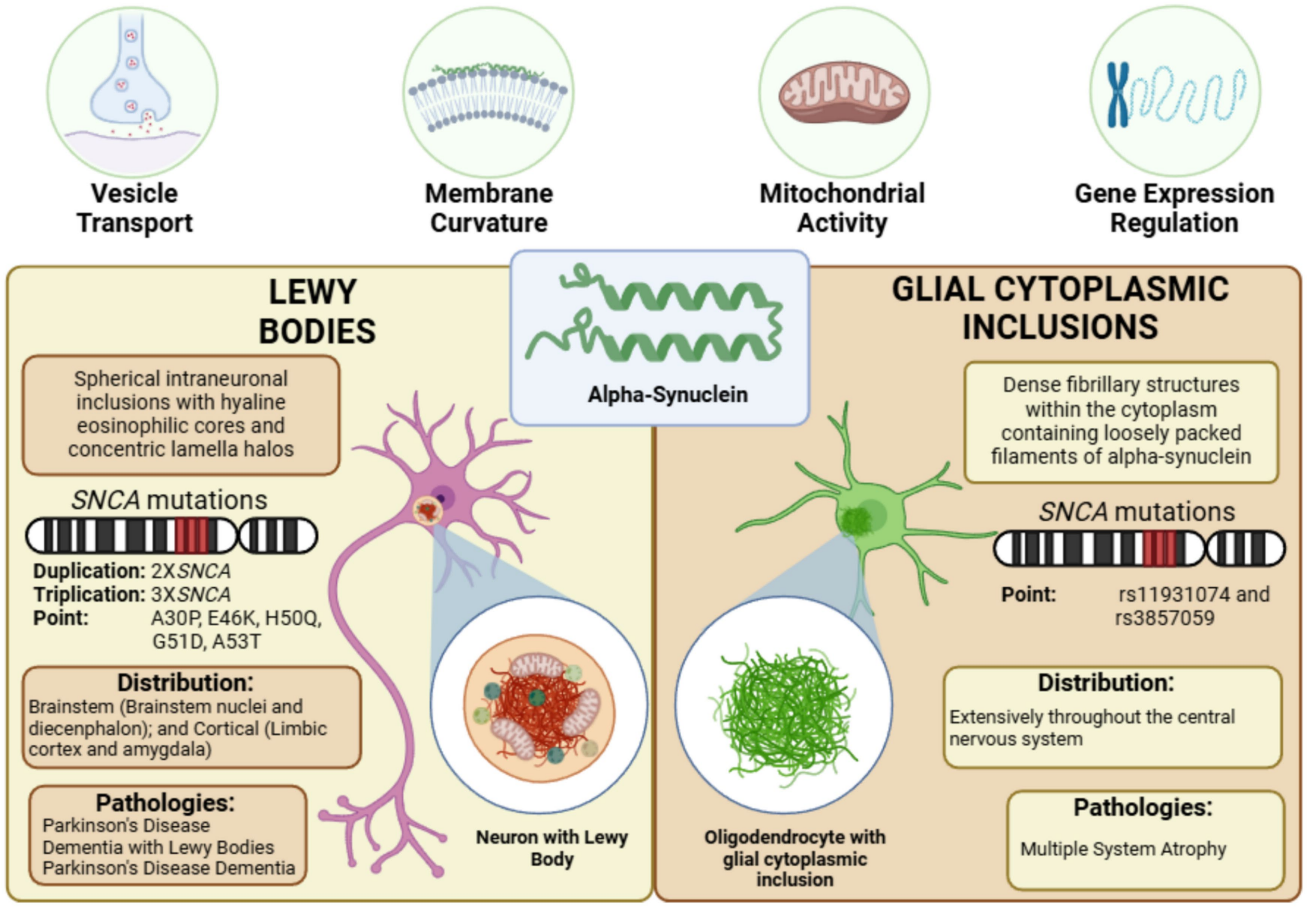
α-Synuclein aggregates in synucleinopathies. α-synuclein misfolds and aggregates into two distinct pathological structures: Lewy bodies (LBs), primarily composed of synphilin-1, ubiquitin-proteasome components, and cytoskeletal proteins, distributed across the brainstem and cortex in various synucleinopathies, and glial cytoplasmic inclusions (GCIs), predominantly formed of p25α/TPPP, ubiquitin-proteasome components, and cytoskeletal proteins, characteristic of multiple system atrophy—figure created with BioRender.com.

Two primary α-syn aggregate forms have been identified: LBs and GCIs (Figure 2). The specific mechanisms underlying the formation of these aggregates and their pathological impact on neuronal and glial function require further elucidation, as they are crucial for understanding the clinical heterogeneity of synucleinopathies.

### 2.3 Lewy bodies

#### 2.3.1 Composition

LBs are the most prevalent α-syn aggregates, representing a hallmark of synucleinopathies. Morphologically, LBs can be classified into two main types: brainstem LBs and cortical LBs. Brainstem LBs are localized within brainstem nuclei and the diencephalon, while cortical LBs are primarily found in the limbic cortex and amygdala. Classic brainstem LBs exhibit spherical intraneuronal inclusions with hyaline eosinophilic cores, concentric lamellar bands, and immunoreactivity for α-syn and ubiquitin, typically surrounded by narrow pale halos (Campbell et al., 2001). In contrast, cortical LBs lack this distinctive halo structure (Gómez-Tortosa et al., 2000). Immunolabeling studies reveal that approximately 64% of nigral LBs and 31% of cortical LBs in PD brains display α-syn immunoreactivity, suggesting regional differences in aggregation profiles (Sakamoto et al., 2002).

The precise biochemical composition of LBs remains incompletely characterized despite identifying over 76 distinct protein components spanning ten functional classes. These include structural proteins, α-syn- and synphilin-1-binding proteins, ubiquitin-proteasome system components, and proteins related to cytoskeletal structure, cellular responses, cell cycle, and signaling pathways (Beyer and Ariza, 2007; Wakabayashi et al., 2007). Notable proteins identified within LBs include Parkin, UCH-L1, and PINK1, ubiquitylated during LB formation (Junn et al., 2002; Muqit et al., 2006). Ubiquitylation, modulated by phosphorylation, is pivotal in the generation of LBs. For instance, α-syn phosphorylation at serine 129 is a hallmark of LBs (Hasegawa et al., 2002; Anderson et al., 2006). Kinases such as GSK3β and Cdk5, which influence dopaminergic neuron survival in pharmacological models of PD, also regulate LB formation. Cdk5 phosphorylates Parkin at serine 131, impairing its ubiquitylation capacity for synphilin-1. Similarly, GSK3β and CKII modulate synphilin-1 phosphorylation, affecting inclusion body formation (Smith et al., 2003; Chen et al., 2004; Chung et al., 2004; Lee G. et al., 2004; Avraham et al., 2005, 2007).

Proteomic analyses reveal that all proteins associated with PD via genetic mutations, except beta-synuclein, are present in LBs. This includes mitochondrial proteins (e.g., PINK1, Parkin, DJ-1, HtrA2/Omi) and proteasome-associated proteins (e.g., UCH-L1, Parkin, synphilin-1). The absence of beta-synuclein from LBs may reflect its anti-aggregatory properties toward misfolded α-syn. However, further comprehensive studies of LB composition across disease stages could illuminate the dysfunction of molecular pathways underlying LB-related neurodegenerative diseases, offering critical insights into their pathogenesis.

#### 2.3.2 Disorders associated with Lewy bodies

##### 2.3.2.1 Parkinson’s disease

LBs are a histopathological hallmark of PD, as first described by Friederich H. Lewy in the dorsal vagal nucleus and nucleus basalis of Meynert (Lewy, 1912) and later confirmed in the substantia nigra by Tretiakoff (1919). Braak et al. (2003, 2006) introduced a staging system outlining the progressive spread of PD α-syn pathology. This staging begins in the dorsal vagal nucleus and olfactory bulb (*stage 1*), advances through the pontine tegmentum (*stage 2*), midbrain and neostriatum (*stage 3*), basal prosencephalon and mesocortex (*stage 4*), and ultimately reaches the neocortex (*stages 5 and 6*).

Although LBs are strongly associated with neurodegeneration in PD, their exact role remains debated. Several observations support their involvement in neuronal loss: significant neuron loss occurs in LB-prone regions such as the substantia nigra, locus coeruleus, and nucleus basalis of Meynert (Björklund and Barker, 2024). Moreover, neurons containing LBs are more abundant in cases with mild to moderate substantia nigra degeneration than in those with severe neuronal depletion, suggesting that LB-bearing neurons may be undergoing cell death (Wakabayashi et al., 2006). Additionally, cortical LB density correlates with cognitive impairment in PD (Wakabayashi et al., 1998b), and LBs may impair axonal transport, contributing to neuronal dysfunction (Katsuse et al., 2003). However, the association of LBs with neuronal loss does not confirm them as a direct cause of cell death (Terry, 2000).

Emerging evidence suggests that LBs may be protective by sequestering toxic proteins and mitigating cellular damage, similar to mechanisms proposed in other neurodegenerative disorders (Tompkins and Hill, 1997; Tanaka et al., 2004). Indeed, recent studies have shown that α-syn oligomers and protofibrils, rather than fibrillar aggregates, are the primary cytotoxic species in PD. Fibrillar aggregates, such as those found in LBs, may serve a protective role by sequestering toxic α-syn species. For example, increased α-syn fibrillation is associated with reduced toxicity in a Drosophila PD model (Chen and Feany, 2005), and proteasome inhibition induces α-syn inclusions while preventing dopaminergic neuron death in rats (Sawada et al., 2004). These findings support the hypothesis that LB formation is an adaptive response to reduce cellular toxicity.

Tanji et al. identified autophagosomal proteins, such as LC3, GABARAP, and HDAC6, in LBs, suggesting that they facilitate autophagy and assist in the degradation of aggregated proteins. Autophagic adapter proteins, including p62 and NBR1, are present in LBs and pale bodies, where they likely sequester toxic oligomeric α-syn (Johansen and Lamark, 2011). These findings suggest that fibrillar α-syn aggregates in LBs may mitigate the toxicity of non-fibrillar forms, highlighting the complex interplay between proteostasis and neurodegeneration in PD.

The relationship between α-syn aggregation and neuronal metabolism also reveals potential protective mechanisms. Mori et al. demonstrated that reduced tyrosine hydroxylase (TH) immunoreactivity in the substantia nigra and locus coeruleus is associated with α-syn aggregation in PD (Mori et al., 2006). Lower TH activity reduces cytotoxic substances such as free dopamine, dopamine quinones, and reactive oxygen species, potentially decreasing the formation of toxic α-syn oligomers. This reduction in dopamine synthesis may thus represent an adaptive mechanism limiting PD neurotoxicity (Sidhu et al., 2004). Together, these findings underscore the dual role of LBs in PD as markers of pathology and potential modulators of cellular stress responses.

##### 2.3.2.2 Lewy body dementia

Although LBs are a hallmark of PD, they are not exclusive. LBs are present in several related pathologies, collectively referred to as LBD. This term has replaced earlier diagnostic labels such as Diffuse Lewy Body Disease (DLBD) (Kosaka et al., 1984; Dickson et al., 1987; Lennox et al., 1989), Cortical Lewy Body Dementia (CLBD) (Byrne et al., 1991), the Lewy Body Variant of Alzheimer’s Disease (LBVAD) (Hansen et al., 1990; Förstl et al., 1993), and Senile Dementia of the Lewy Body Type (SDLT) (Perry et al., 1990). The classification of LBD arose from post-mortem neuropathological findings, which revealed characteristic distributions of LBs and Lewy neurites in elderly patients with dementia. Retrospective clinical analyses of these cases highlighted two significant observations (McKeith, 2017):

- Many patients with LB pathology exhibited clinical symptoms distinct from other forms of dementia, even in the early stages. These core features include cognitive fluctuations, recurrent visual hallucinations, spontaneous extrapyramidal motor signs, and a history of REM sleep behavior disorder. A probable diagnosis of LBD is supported by the presence of at least two of these features in a patient with dementia.
- Approximately 50% of cases with significant LB pathology lacked typical LBD symptoms during life, instead presenting with global cognitive decline similar to Alzheimer’s disease (AD). Such cases often show considerable AD-related neuropathological changes (Montine et al., 2012; Tiraboschi et al., 2015). Notably, autopsy studies indicate that between one-third and one-half of clinically diagnosed AD cases exhibit some degree of LB pathology (Toledo et al., 2013). Complex visual hallucinations are the sole clinical feature consistently associated with LB pathology in otherwise typical AD cases (Thomas et al., 2018), although data on progression, prognosis, and treatment responses in mixed AD+LBD cases remain limited.

The underdiagnosis of LBD during life remains a significant clinical challenge. A study of specialist dementia referrals found that only 4.6% of patients were diagnosed with LBD, yet post-mortem analyses revealed LB pathology in approximately 20% of cases. Diagnostic rates among clinicians varied significantly (2.4–5.9%) even within nearby regions, highlighting the need for improved diagnostic approaches, including integrating biomarkers (Kane et al., 2018). Parkinsonism further complicates diagnosis, as motor features are mild or absent in up to 25% of confirmed LBD cases. Diagnostic criteria require at least one motor feature (e.g., bradykinesia, resting tremor, or rigidity) for an LBD diagnosis, whereas PD diagnosis requires two. Coexisting conditions such as arthritis or cognitive impairment can further obscure the clinical presentation.

Recurrent, complex visual hallucinations are a hallmark of LBD and are relatively easy to recognize when clinicians inquire directly and use validated severity scales. These hallucinations often involve vivid, well-formed images of people or animals and may be accompanied by illusions or a sense of presence. Caregivers and patients can generally report these phenomena (Perry et al., 1990). Consensus diagnostic criteria for probable LBD have an estimated autopsy specificity of ~85%, one of the highest among neurodegenerative dementias. Incorporating biomarkers may further improve diagnostic accuracy, though their potential impact is still under investigation (Rizzo et al., 2018). Persistent or combined supportive clinical features could also enhance diagnostic confidence.

##### 2.3.2.3 Parkinson’s disease dementia

PDD represents another type of LB-related pathology occurring in patients with PD who develop cognitive decline. The distinction between PDD and LBD remains a subject of ongoing debate, as both conditions share overlapping neuropathological features in advanced stages. However, their clinical trajectories differ markedly. Early cognitive impairments and relatively mild motor symptoms typically characterize LBD, whereas prominent early motor features followed by the later onset of cognitive and neuropsychiatric symptoms define PDD. Clinicians formally distinguish these conditions using the ‘one-year rule’: LBD is diagnosed when dementia emerges before or concurrently with parkinsonism, whereas PDD is diagnosed when dementia develops after well-established PD. The DSM-5 (American Psychiatric Association, 2013) and ICD-11 (Emanuele et al., 2016) endorse this criterion.

The mean age of onset for both LBD and PDD exceeds 70 years, contrasting with the earlier onset of motor symptoms in PD, which typically occurs around 60 years. Studies comparing the prevalence and onset ages of LBD and PDD have yielded mixed findings. While some reports suggest an earlier onset of symptoms in LBD, others find no significant differences or indicate that PDD may develop at a younger age (Jellinger and Korczyn, 2018). These discrepancies underscore the need for further research to delineate the clinical and pathological boundaries between these LB-associated conditions.

### 2.4 Glial cytoplasmic inclusions

#### 2.4.1 Composition

GCIs represent a distinct form of α-syn aggregation, predominantly found in oligodendrocytes and playing a critical role in the pathogenesis of MSA. Unlike LBs, which are localized in neurons, GCIs are confined to glial cells, where oligodendroglial dysfunction contributes to disease progression (Papp et al., 1989). Approximately 50% of non-α-syn-expressing oligodendrocytes in pontine fiber tracts of brains with GCIs exhibit abnormal accumulation of TPPP/p25α, accompanied by increased cell size (Song et al., 2007). TPPP/p25α, an oligodendroglial-specific phosphoprotein, typically colocalizes with myelin essential protein (MBP) in healthy brains, promotes α-syn polymerization and is a prominent GCI component. However, in MSA, the association between TPPP/p25α and MBP is disrupted (Lindersson et al., 2005; Song et al., 2007).

Autophagy-related pathways are also implicated in GCI formation. GCIs contain the autophagy-associated protein AMBRA1, which mildly reduces abnormal α-syn levels in co-transfection models. In MSA brains, AMBRA1 levels are elevated, while TNF receptor-associated factor 6, an upstream regulator of autophagy, is decreased, indicating dysregulation of these pathways (Miki et al., 2018). This evidence suggests a complex relationship between autophagy dysfunction and α-syn accumulation in oligodendrocytes, although the sequence of events leading to inclusion formation remains uncertain.

#### 2.4.2 Disorder with glial cytoplasmic inclusions

MSA is a fatal, progressive neurodegenerative disorder characterized by autonomic dysfunction, parkinsonian features, cerebellar ataxia, and corticospinal tract degeneration. Symptoms include tremor, rigidity, bradykinesia, coordination deficits, and autonomic disturbances such as bladder dysfunction, orthostatic hypotension, and fainting (O’Sullivan et al., 2008; Fanciulli and Wenning, 2015; Fanciulli et al., 2019; Poewe et al., 2022). Classified as a synucleinopathy, MSA’s defining neuropathological feature is the presence of GCIs in oligodendrocytes (Papp et al., 1989; Tu et al., 1998; Armstrong et al., 2006).

The pathogenesis of MSA remains poorly understood, but prevailing theories implicate excessive α-syn production, increased uptake by neurons and oligodendrocytes, impaired α-syn degradation through autophagic and proteasomal pathways, mitochondrial dysfunction, oxidative stress, and neuroinflammation (Monzio Compagnoni and Di Fonzo, 2019). While MSA is generally sporadic, its heritability is estimated between 2.09 and 6.65%, with rare familial cases reported in autosomal dominant and autosomal recessive patterns (Wullner, 2004; Vidal et al., 2010; Itoh et al., 2014).

GCIs, the hallmark lesion of MSA, are argyrophilic inclusions derived from oligodendrocytes (Wakabayashi and Takahashi, 2006). Although α-syn is primarily expressed in neurons, mRNA and protein have been detected in oligodendrocytes, suggesting that α-syn overexpression in these cells contributes to GCI formation (Richter-Landsberg et al., 2000; Mori et al., 2002). Evidence indicates that GCIs appear prior to neuronal degeneration, as demonstrated in “minimal change” MSA cases where neuronal loss is limited to the striatonigral system, yet GCIs are extensively distributed throughout the CNS (Inoue et al., 1997; Inoue et al., 1997; Mochizuki et al., 2002; Ling et al., 2015). The frequency of GCIs correlates with the severity of neuronal loss in the striatonigral system, implicating these inclusions in disease progression (Nakazato and Suzuki, 1996; Ozawa, 2004).

Myelin degradation is a key pathological feature of MSA, with evidence suggesting that myelin loss in the pontine base precedes axonal loss (Wakabayashi et al., 2005). Apoptosis predominantly affects oligodendrocytes, and significant myelin damage is evident in MSA brains (Matsuo et al., 1998; Probst-Cousin et al., 1998; Wakabayashi et al., 1998a). This supports the hypothesis that widespread GCI formation drives oligodendroglial degeneration and myelin autophagy, contributing to CNS dysfunction.

Animal models have provided significant insights into MSA pathogenesis. Transgenic mice overexpressing α-syn in oligodendrocytes under the myelin essential protein promoter exhibit progressive motor impairment, dopaminergic fiber loss, and filamentous aggregates associated with myelin autophagy and cell loss (Zuscik et al., 2000, 2001; Papay et al., 2002; Shults et al., 2005; Stefanova et al., 2005; Yazawa et al., 2005; Stefanova et al., 2005; Yazawa et al., 2005; Shults et al., 2005). Other models combining oligodendroglial α-syn overexpression with mitochondrial dysfunction recapitulate MSA-like pathology, highlighting the interplay between oligodendroglial degeneration and α-syn aggregation as central to disease progression (Stefanova et al., 2005). These studies underscore the critical role of GCIs and oligodendroglial dysfunction in driving neurodegeneration in MSA.

### 2.5 Protein heterogeneity

Although LBs and GCIs share α-syn as their primary aggregating protein and are pathological hallmarks of synucleinopathies, they exhibit distinct structural and molecular characteristics. GCIs, predominantly found in oligodendrocytes, are composed of dense, filamentous aggregates within the cytoplasm. In contrast, LBs are located in neurons and are characterized by concentric eosinophilic cores surrounded by a halo of radiating filaments. However, the most pronounced differences between LBs and GCIs lie in their protein composition (Table 1).

**Table 1:** Protein composition of α-synuclein aggregates and associated proteinopathies.

Protein	Typically associated proteinopathies	GCIs	LBs	References
Chaperons				
α-syn	PD, LBD, MSA, PAF	+	+	Spillantini et al. (1997) and Kaji et al. (2020)
Heat shock protein 70 and 90	PD, HD, ALS, AD	+	+	Uryu et al. (2006), Kawamoto et al. (2007), Chiba et al. (2012), and Gupta et al. (2020)
DJ-1	PD	+	–	Neumann et al. (2004)
α/β-crystallin	AD, PiD, AlD	+	+/–	Lowe et al. (1992a,b) and Murayama et al. (1992)
Cytoskeletal proteins				
α/β-tubulin	PD, AD, HD	+	+	Galloway et al. (1988), Kato and Nakamura (1990), Abe et al. (1992), and Mazzetti et al. (2024)
Tau (non-phosphorylated)	AD, PiD, CBD, PSP	+/–	+/–	Galloway et al. (1989), Kato and Nakamura (1990), Murayama et al. (1992), Ishizawa et al. (2003), Lewczuk et al. (2016), and Goossens et al. (2017)
Tau (phosphorylated)	AD, PiD, CBD, SPS, AGD	–	+/–	Cairns et al. (1997), Kuusisto et al. (2001), and Ishizawa et al. (2003)
Microtubule-associated protein 1	AD, PD, ALS	+/–	+	Galloway et al. (1988), Abe et al. (1992), and Dubey et al. (2015)
Microtubule-associated protein 2	HD	–	+	Galloway et al. (1988), Abe et al. (1992), Arima et al. (1992), and DeGiosio et al. (2022)
TPPP/p25 (tubulin polymerization-promoting protein)	PD, MSA	+	+	Kovács et al. (2004), Song et al. (2007), Yoshida (2011), and Oláh and Ovádi (2019)
Ubiquitin and autophagy-related proteins				
Ubiquitin	PD, AD, HD, ALS	+	+	Lowe et al. (1988), Murayama et al. (1992), Atkin and Paulson (2014)
SUMO-1 (small ubiquitin modifier 1)	AD, HD, PSP	+	+/–	Pountney et al. (2005), Kim et al. (2011), and Knock et al. (2018)
20s proteasome subunits	PD, AD, HD	+	–	Olanow et al. (2004), Chiba et al. (2012), and Fernández-Cruz and Reynaud (2021)
HDAC6	AD, PD, HD, ALS	+	+	Miki et al. (2011) and Van Helleputte et al. (2014)
Parkin	PD, AD, ALS, HD	+/–	+	Murakami et al. (2004), Huang et al. (2008), and Zhang et al. (2016)
Pael-R	PD, AR-JP	–	+	Murakami et al. (2004) and Zou et al. (2012)
Dorfin	ALS, PD	+	+	Hishikawa et al. (2003) and Ito et al. (2003)
NEDD-8	PD, AD	+	+	Mori et al. (2005)
NUB1 (Negative regulator of ubiquitin-like protein 1)	PD, LBD, MSA	+	+	Tanji et al. (2007, 2019)
Synphilin-1	PD	+	+	Engelender et al. (1999) and Wakabayashi et al. (2000, 2002)
F-box only protein (FBXO7)	PD, PPS	+	+	Zhao et al. (2013)
p62/SQSTM1	AD, PD, HD	+	+	Kuusisto et al. (2001) and Liu et al. (2017)
LC3	AD, PD	+	+	Yue et al. (2009), Tanji et al. (2011, 2013), and Heckmann et al. (2019)
NBR1	HD, PD	+	+	Odagiri et al. (2012) and Park et al. (2020)
AMBRA1	PD	+	+	Miki et al. (2016, 2018) and Di Rita et al. (2018)
Apoptosis regulators				
Bcl-2	AD, HD, PD, ALS	+	+	Probst-Cousin et al. (1998) and Shacka and Roth (2005)
HtrA2/Omi	PD	+	+	Kawamoto et al. (2008) and Vande Walle et al. (2008)
Parkin co-regulated gene (PACBG)	PD	+	+	Taylor et al. (2007)
XIAP (X-linked inhibitor of apoptosis protein)	PD, LBD	+	+	Kawamoto et al. (2012, 2014)
Apoptosome (cytochrome c, Apaf-1, caspase-9)	AD, PD, HD, ALS	+	+	Friedlander (2003) and Kawamoto et al. (2012, 2014)

+, positive; +/–, partially or weakly positive; –, negative; AD, Alzheimer’s Disease; AGD, Argyrophilic Grain Disease; AlD, Alexander’s Disease; ALS, Amyotrophic Lateral Sclerosis; AR-JP, Autosomal Recessive Juvenile Parkinsonism; CBD, Corticobasal Degeneration; HD, Huntington’s Disease; LBD, Lewy Body Dementia; MSA, Multi-System Atrophy; ND, not described; PAF, Pure Autonomic Failure; PD, Parkinson’s disease; PiD, Pick’s Disease; PSP, Progressive Supranuclear Palsy; PPS, Parkinsonian-Pyramidal Syndrome. Modified from Kaji et al. (2020).

Quantitative immunomagnetic analysis by McCormack et al. revealed that GCIs consist of 11.7% α-syn, 1.9% αβ-crystallin, and 2.3% 14–3-3 proteins, compared to 8.5, 2.0, and 1.5% in LBs, respectively (McCormack et al., 2016). This emphasizes the predominance of fibrillary α-syn in GCIs and its central role in their formation. Despite these differences, LBs and GCIs share many common components, including cytoskeletal proteins, molecular chaperones, aggresomal proteins, and apoptosis mediators. However, GCI-specific constituents, such as midkine, Leu-7, transferrin, and TPPP/p25α, suggest a distinct oligodendrocyte-specific role in GCI pathogenesis (Wakabayashi et al., 2007; Jellinger, 2018).

The α-syn aggregates in GCIs exhibit relatively low levels of phosphorylated α-syn and high affinity for the monoclonal antibody Syn7015, which preferentially identifies a distinct α-syn species with limited ability to cross-seed tau aggregation (Guo et al., 2013; Peng et al., 2018). This implies a weak interaction between GCI-α-syn and tau, typically confined to neuronal axons and rarely observed in glial cells under normal conditions. Post-translational modifications further differentiate the two aggregates; for example, NUB1 (NEDD8 ultimate buster 1) colocalizes with both LBs and GCIs, but its phosphorylation at S46—associated with aggregate degradation—is observed only in LBs (Tanji et al., 2007, 2019).

These compositional and post-translational differences reflect the influence of the cellular environment on the formation of LBs and GCIs, as well as the distinct pathological mechanisms they drive. The oligodendrocyte-specific environment of GCIs implicates disrupted myelination and oligodendrocyte death as contributors to axonal degeneration in MSA. Conversely, the broader protein composition of LBs underscores disrupted neuronal proteostasis and mitochondrial dysfunction, central features of synucleinopathies such as PD. These differences enhance our understanding of disease mechanisms and present opportunities for therapeutic strategies tailored to each pathology.

Despite significant advancements, most research on α-syn aggregation has focused on its behavior as an isolated entity, with limited exploration of its interactions with other proteins. This is noteworthy given the presence of proteins such as tau—primarily associated with AD—in LBs and GCIs. The co-occurrence of tau and α-syn pathology has been observed in various synucleinopathies, including LBD and PDD (Table 1), suggesting a synergistic relationship that may exacerbate neurodegeneration. Studying tau within the context of synucleinopathies could provide insights into their physiological and pathological interactions, offering a broader perspective on their roles in disease mechanisms. Understanding these interrelationships may elucidate how aggregation processes interact, driving the progression of neurodegenerative diseases like Huntington’s disease (HD), amyotrophic lateral sclerosis (ALS), AD, LBD, and others (Table 1).

## 3 Tau in synucleinopathies

### 3.1 Physiological roles of tau

As with α-syn, a thorough understanding of tau begins with its physiological roles, which precede the pathological processes underlying its aggregation. Tau is a highly soluble protein encoded by the *MAPT* (microtubule-associated protein tau) gene, produced via alternative splicing of its pre-mRNA that derives into six isoforms of the protein in humans (Goedert et al., 1989). Its primary role is maintaining microtubule stability in axons, a capacity directly related to its structure and microtubule-binding sites. The six human isoforms of tau differ from each other by the presence or absence of one or two inserts in the N-terminal part and by the presence of either three or four microtubule-binding repeats (R) in the C-terminal half, named 3R-tau and 4R-tau (Andreadis et al., 1992). Furthermore, tau is broadly expressed throughout the brain, predominantly in neurons and astrocytes, though small amounts are detectable in non-neuronal cells, including oligodendrocytes in rat brains (Binder et al., 1985; Shin et al., 1991; LoPresti et al., 1995; LoPresti, 2018). Tau’s subcellular distribution shifts during development: it is uniformly present in young neurons’ cell bodies and neurites but localizes predominantly to axons as neurons polarize, with significantly reduced levels in dendrites and nuclei (Papasozomenos and Binder, 1987; Iwata et al., 2019). Additionally, tau has been observed in the synaptic compartments of healthy neurons, contributing to synaptic physiology (Regan et al., 2017; Robbins et al., 2021).

In adult neurons, axonal tau is crucial for polymerizing and stabilizing microtubules. As traditionally viewed, phosphorylated tau binds to the C-terminus of tubulin, promoting microtubule assembly and supporting the neural cytoskeleton (Cleveland et al., 1977a; El Mammeri et al., 2022). However, recent studies have reported tau to be concentrated in the labile domains of axonal microtubules, suggesting a change in paradigm regarding its primary role based on extending these domains rather than stabilizing them (Kent et al., 2020; Cario and Berger, 2023). Furthermore, axonal tau facilitates cytoskeletal reorganization, morphological definition, and axonal elongation. Knockdown experiments have demonstrated that tau is essential for neurite formation in cultured rat neurons, while overexpression promotes neurite outgrowth, though this effect has shown variability across different cell types (Knops et al., 1991; Caceres et al., 1992; Dawson et al., 2001; Wang and Mandelkow, 2016).

Tau is also detected in dendrites and possibly in dendritic spines, though its postsynaptic functions remain debated due to ambiguities in synaptosome studies (Ittner et al., 2010; Mondragón-Rodríguez et al., 2012; Frandemiche et al., 2014; Tai et al., 2014; Yin et al., 2021). Evidence suggests that tau may influence synaptic plasticity; for instance, tau translocates from dendritic shafts to excitatory postsynaptic sites in response to pharmacological activation in cultured neurons and hippocampal slices (Frandemiche et al., 2014). Tau interacts with the Src kinase Fyn to mediate postsynaptic signaling, and tau knockout mice exhibit abnormalities in synaptic signal transduction (Ittner et al., 2010).

Beyond its roles in the cytoplasm, tau is present in the nucleus, which plays a role in genomic stability and RNA regulation. Dephosphorylated tau maintains the integrity of genomic DNA under physiological and hyperthermic stress conditions, while phosphorylated tau disrupts nucleocytoplasmic transport and induces nuclear envelope invagination (Violet et al., 2014; Bukar Maina et al., 2016; Cornelison et al., 2019). Hyperphosphorylated tau binds the nuclear pore complex components, impairing nucleocytoplasmic transport (Tripathi et al., 2019). Studies in tau-transgenic Drosophila have shown cell death associated with polyadenylated RNA accumulation around tau-induced nuclear envelope invaginations (Cornelison et al., 2019). Mutations in the *MAPT* gene further disrupt nuclear transport processes (Paonessa et al., 2019). Nuclear tau has also been observed in human fibroblast and neuroblastoma cells (Loomis et al., 1990; Ibáñez-Salazar et al., 2017).

Post-translational modifications, such as phosphorylation and acetylation, regulate tau’s subcellular localization and interactions. Tau isoforms lacking exons 2 and 3 efficiently target axons, while the 2N4R isoform is partially retained in the soma and dendrites (Zempel et al., 2017; Bachmann et al., 2021). These modifications also influence tau’s interaction with membranes, increasing its presence in dendrites under physiological and pathological conditions (Arrasate et al., 2000; Sallaberry et al., 2021; Llorens-Martin et al., 2014). These findings highlight the complexity of tau’s physiological roles, which span microtubule stabilization, synaptic plasticity, and nuclear functions, and underscore how disruptions in these roles can contribute to pathological processes.

### 3.2 Preservation of proteostasis in tau

As well as α-syn, tau is primarily degraded through the UPS and the ALP (Bednarski and Lynch, 1996). The UPS is responsible for degrading soluble tau, with studies showing that both 20S and 26S proteasomes can break it down, either directly or following ubiquitylation (Inoue et al., 2012). Proteasomal inhibitors like MG-132 and lactacystin lead to tau accumulation in certain cell models, but proteasomal inhibition does not always increase tau levels, suggesting that ALP may compensate when UPS function is impaired (Inoue et al., 2012). The ALP primarily clears aggregated or hyperphosphorylated tau, with cathepsin D playing a key role in tau degradation. Impairment of lysosomal function, as seen with chloroquine treatment, increases tau levels while activating autophagy with agents like methylene blue, trehalose, or rapamycin enhances tau clearance (Krüger et al., 2012). Genetic studies further confirm ALP’s importance, as knocking out Atg7 in mouse models results in phosphorylated tau accumulation and neurodegeneration (Inoue et al., 2012). Interestingly, UPS inhibition can sometimes trigger autophagy, highlighting the dynamic interplay between these two pathways in maintaining tau homeostasis (Krüger et al., 2012). Notably, as for α-syn, disruption of the proteostasis mechanisms derives in tau misfolding and aggregation.

### 3.3 Tauopathies: pathological mechanisms

The primary isoform of human tau spans 441 amino acids and contains 79 serine/threonine residues, of which 20 phosphorylation sites are linked to its functionality (Sergeant et al., 2008). Tau’s physiological roles are regulated by phosphorylation and dephosphorylation, which modulate its interaction with microtubules and influence its filament-forming capacity by altering regions within acidic N-terminal domains (Alonso et al., 2001). Notably, tau hyperphosphorylation correlates with AD severity, as demonstrated by the development of tau-specific phosphorylation markers that link tau accumulation, particularly in paired helical filaments, to disease progression (Augustinack et al., 2002).

Early stages of tau aggregation involve phosphorylation at KXGS motifs in the microtubule-binding region, particularly at Ser262, a modification observed in normal adult brains that likely reduces tau’s microtubule affinity and regulates its aggregation potential (Watanabe et al., 1993; Preuss et al., 1997; Gärtner et al., 1998). Phosphorylation at T231, another early marker, appears in pre-tangle tau aggregates, exhibiting reduced microtubule affinity but also occurring in non-disease brains (Watanabe et al., 1993; Luna-Muñoz et al., 2007). Hyperphosphorylation at additional sites, including pS199, pS202, pT205, pS208, pS396, and pS404, is commonly observed in tauopathies and is thought to promote aggregation and disease progression (Otvos et al., 1994; Rankin et al., 2005, 2007; Li and Paudel, 2006; Porzig et al., 2007), although further research is needed to confirm this assertion.

Familial tau mutations, such as *G272V*, *P301L*, and *V337M*, enhance phosphorylation and tau aggregation, whereas the R406W mutation reduces phosphorylation at key residues (T231, S235, S396, S400, and S404) (Connell et al., 2001). These mutations alter tau’s conformation, facilitating aggregation and recruitment of microtubule-associated tau, further highlighting the role of phosphorylation in tau pathology (Alonso et al., 2004; Han et al., 2009).

Dephosphorylation, in contrast, enhances tau’s microtubule-binding affinity, while phosphorylation reduces this interaction, promoting tau’s dissociation from microtubules and subsequent aggregation (Bramblett et al., 1993; Poppek et al., 2006). In physiological brains, tau phosphorylation at residues such as S199, S202, T231, S262, and S404 prepares it for interactions with other proteins (Watanabe et al., 1993; Liu et al., 2010). However, hyperphosphorylation in disease states leads to tau misfolding, forming insoluble paired helical filaments that aggregate into neurofibrillary tangles (NFTs) within neurons.

Three major kinase classes contribute to tau phosphorylation: proline-directed kinases (e.g., GSK3β, ERK1/2, JNK, p38, CDK5), non-proline-directed kinases (e.g., tau-tubulin kinase, MARKs, casein kinase, DYRK1A/2), and tyrosine kinases (e.g., Src, c-Abl). Tyrosine-phosphorylated tau is particularly notable, as it has been identified in aggregates in transgenic models and AD brains, suggesting its potential contribution to tau aggregation and its value as a drug target (Vega et al., 2005; Lee et al., 2011; Martin et al., 2013). Hyperphosphorylation, especially in KXGS motifs, predisposes tau to aggregation. Although unphosphorylated tau can polymerize *in vitro*, hyperphosphorylation neutralizes anti-aggregation regions, promoting filament formation by altering tau’s local structure and charge distribution (Crowther et al., 1994; Alonso et al., 2001; Haase et al., 2004; Dolai et al., 2011).

Beyond phosphorylation, other post-translational modifications (PTMs), such as acetylation, proteolytic cleavage, nitration, glycosylation, and glycation, also influence tau aggregation. Acetylation, first identified by Min et al., is proposed as an initial step in accumulating phosphorylated tau in NFTs (Min et al., 2010). However, conflicting evidence suggests tau may be hypoacetylated yet hyperphosphorylated in AD brains and tau transgenic models, particularly at KXGS motifs (Cook et al., 2014). Proteolytic cleavage by caspases 3 and 6 at D421 and D348, especially D421-truncated tau, significantly accelerates aggregation and may be an early step in NFT formation (Ugolini et al., 1997; Gamblin et al., 2003; Rissman et al., 2004; Cotman et al., 2005; Basurto-Islas et al., 2008). Among truncated fragments, Tau151-391 has been identified as the most pathogenic (Gu et al., 2020). Other modifications, including glycosylation, glycation, and nitration, have been associated with tauopathy mechanisms (Wang et al., 1996; Nacharaju et al., 1997; Horiguchi et al., 2003; Kuhla et al., 2007).

**Figure 3:**
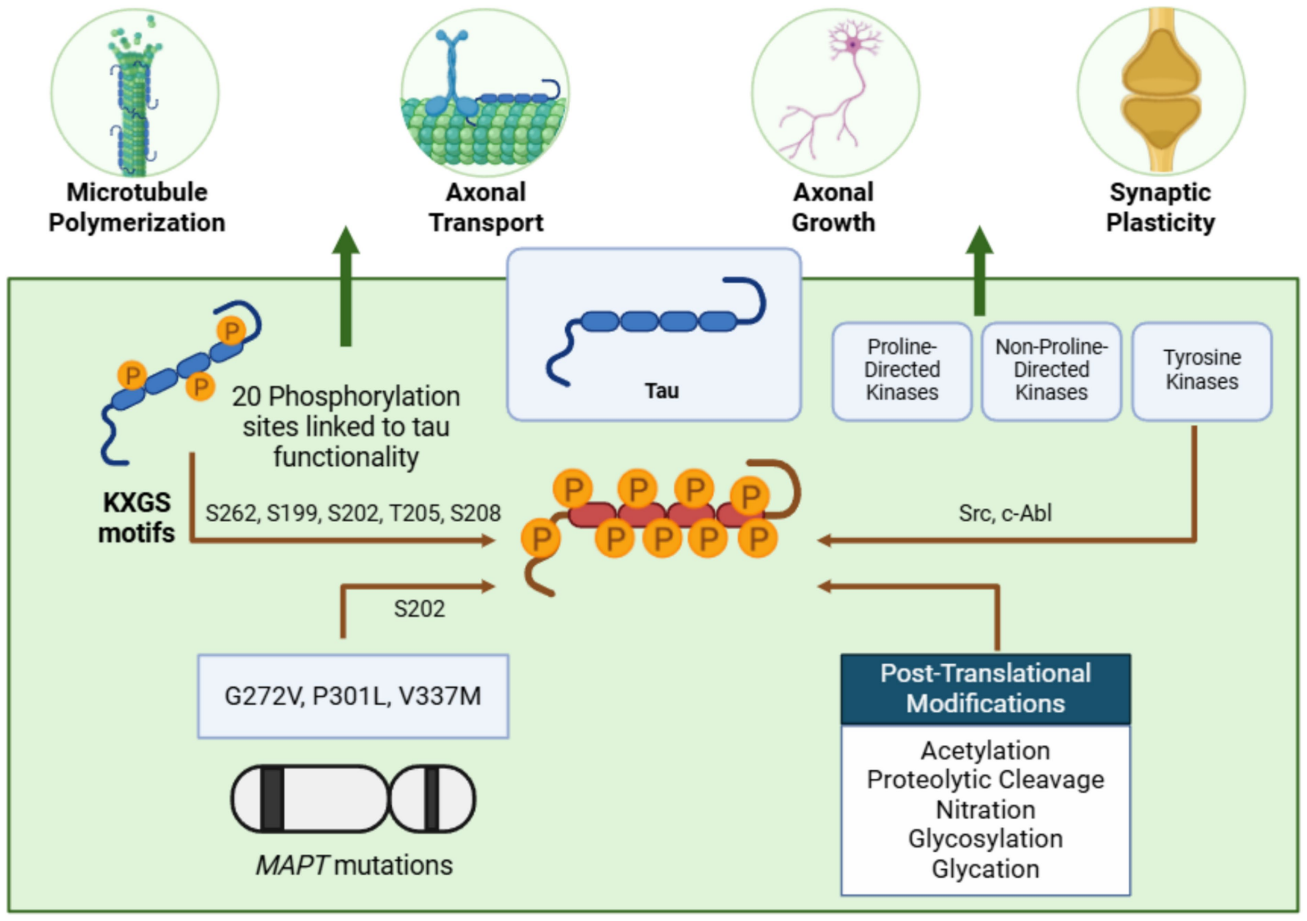
Tau: a microtubule stabilizer prone to pathological aggregation. Tau stabilizes microtubules and supports critical neuronal processes such as axonal transport, growth, and synaptic plasticity. Phosphorylation at KXGS motifs, mediated by tyrosine kinases, regulates its function. However, mutations in the *MAPT* gene, alterations in these motifs, and post-translational modifications can lead to tau hyperphosphorylation, reducing its microtubule affinity and promoting aggregation—figure created with BioRender.com.

The interplay of multiple PTMs, often occurring concurrently, underscores the importance of maintaining a precise balance for tau’s normal function. Even minor deviations from this equilibrium may render tau aggregation-prone, contributing to its pathological buildup. These mechanisms collectively illustrate how tau’s molecular alterations drive the progression of tauopathies. The above is summarized in Figure 3.

### 3.4 Evidence of misfolded tau in Parkinson’s disease

While tau misfolding and aggregation are well-characterized in AD, evidence of tau’s presence in α-syn aggregates, such as LBs and GCIs, has shifted the paradigm from isolated proteinopathies to potential interactions between tau and α-syn (Table 2). Early studies by Galloway et al. documented paired helical filaments (PHFs) of tau in the outer rim of LBs in the locus coeruleus and substantia nigra of post-mortem PD brains (Galloway et al., 1988, 1992). Although initial experiments failed to detect tau immunoreactivity with tau-1 antibodies, subsequent ultrastructural studies revealed PHF immunoreactivity in both the core and rim of LBs (Galloway et al., 1988, 1992). These findings highlighted the impact of tissue treatment on epitope accessibility, with variations observed between tris-buffered saline and sodium dodecyl sulfate treatments.

**Table 2:** Tau in Parkinson’s disease post-mortem brains.

Studied structures	Braak stage	Antibodies	Pathological findings	References
Locus coeruleus, Substantia nigra	NA	For tau: AD-PHF (isoform 5-25, 3-39 and 3-13), PHF (pharmaceutical antibody), PHF homemade antiserums homemade, tau-1 (tau 1 isoform)	LBs were positive to AD-PHF isoform 5-25 and 3-39, PHF and PHF antiserum II; the signal was observed in the outer rim pattern of the LB.	Galloway et al. (1988)
Locus coeruleus, Substantia nigra	NA	PHF antiserum II	The same immunostaining pattern in the nucleus and edge of the LBs. The TBS-treated LBs either had rim-only staining or a pattern identical to intact tissue. Those treated for 36 h had the rim-only staining. SDS-treated LBs were unstained.	Arima et al. (1999)
Locus coeruleus, Dorsal Vagus Nucleus	I & III	For α-syn: PQE3 (NACP/ α-syn) For tau: AT8 [pSer202/Thr205], tau-2 [Ala95, Ala108]	Granular tau-deposits were found around LBs in all cases (PQE3 immunoassays), distinguishing four types: type 1, LBs with ring-shaped tau-immunoreactivity; type 2, LBs surrounded by NFTs; type 3, NACP- and tau-immunoreactive filamentous and granular masses; and type 4, NACP- and tau-immunoreactive dystrophic neurites. In locus coeruleus, all LB types and tau immunoreactive neurites were present. In the dorsal vagus nucleus, LB types 1 and 3 were observed.	Arima et al. (1999)
Amygdala	NA	For α-syn: Syn202 and Syn303 [oxidized α-syn] For tau: AT8, PHF-1 [tau-Ser396/404], 12E8 [pSer262/pSer356], T14 [p141-149] and T46 [p404-441], antiserum 17,026 to recombinant tau	NFTs were variably abundant in the amygdala and the rest of the brain. Tau-positive astroglia were identified in 20 22 PDC cases with highly variable density and morphology. In 7 of 19 patients with PDC, a moderate to high density of LBs and α-syn-positive neurites were identified in the amygdala and showed at least a small number of neurons containing both an NFT and an LB (irregularly shaped and occasionally fragmented) within the same cell. No α-syn-positive pathology was identified in the amygdala of any of the patients except one with AD.	Forman et al. (2002)
Amygdala, Putamen, Caudate, Dorsal pons, Substantia nigra, Medula, Hypothalamus, Lower medulla, Thalamus, Nucleus basalis, Midfrontal cortex, Cingulate, Cerebellum, Hippocampus	NA	For α-syn: Syn 208, Syn 211, Syn 303, LB509, nSyn856 (nitrated α-syn); SNL4, nSyn14 (nitrated α-syn) For tau: T14, T46, PHF-1	LB509 and Syn303 immunoreactivity was present in spheroids and Lewy neurites in multiple brain regions. A cluster of large spheroids with a strongly α-syn-immunoreactive nucleus surrounded by a weak tau-immunoreactive ring was shown. Neuritic pathology was typically positive for either tau or α-syn, but not both proteins. Inclusions containing insoluble tau fibrils were also detected *in situ* using the antibody PHF-1.	Kotzbauer et al. (2004)
Striata	NA	For tau: CP-13 [tau-pSer202] and PHF-1	Western blot showed a 34% increase in p-tau, hyperphosphorylated at pSer262 in PD and PDD. The phosphorylated pSer396/404 form of tau was increased by 81% in PD and 64% in PDD. A significant 23% increase in pSer202 was observed in PD, whereas no change was observed for the PDD group compared to the control group.	Wills et al. (2010)
Cortex (entorhinal and fusiform), Hippocampus, Lateral temporal neocortex	III-VI	For α-syn: clone KM51 For tau: AT8	Mathematical models and diagrams.	Compta et al. (2011)
Cortex, Striata, Insular Cortex, Putamen, Caudate	NA	For tau: AT8	Cortex and striatum: tau in the form of NFTs, neuropil threads, and inclusions. Striatum: α-syn and AT8 co-localization.	Cisbani et al. (2017)
Entorhinal cortex, Amygdala	NA	For tau: tau-pThr175, tau-pThr217, tau-pSer208, tau-pSer210, tau-pThr231, T22 [oligomeric tau], AT8	Glial pathology is present in all regions except the dentate gyrus and substantia nigra (tau-pThr217). Tau pathology observed in all brain regions except the substantia nigra Notably, PHF-tau positive astrocytic plaques were observed in the frontal cortex, while tufted astrocytes were observed in the amygdala.	Moszczynski et al. (2017)
Substantia nigra	NA	For α-syn: pS129 For tau: tau-pT231 (“*cis* p-tau”), AT100, AT8	Both phosphorylated α-syn and tau spread in the substantia nigra, whereas cis P-tau diffused more extensively in PD human brains.	Hadi et al. (2021)
Temporal cortex, Caudate, Putamen, Substantia nigra	II	For tau: tau-pS199/202	Western blot for p-tauS199/202 protein levels was a 4.9-fold increase in PDD compared to DLB.	Tu et al. (2022)

AD, Alzheimer’s disease; AT8, anti-phospho-tau; DLB, Dementia with Lewy bodies; LB, Lewy body; NA, Not applicable; NACP, non-Aβ component precursor; NFT, neurofibrillary tangle; p, phosphorylated; PD, Parkinson’s disease; PDC, Parkinson-dementia complex; PDD, Parkinson’s disease dementia; PHF, paired helical filaments; PQE3, antibody for NACP; SDS, sodium dodecyl sulfate; SNL4, antibody for α-syn; TBS, tris-buffered saline.

Arima et al. later reported the colocalization of phosphorylated tau and α-syn (NACP/α-syn) in LBs across multiple brain regions, including the superior pons, locus coeruleus, and medulla oblongata, in patients with PD and LBD. They classified these inclusions into four types based on immunoreactivity patterns (Arima et al., 1999). Similarly, in the Chamorro ethnic group of Guam, phosphorylated tau was found in the amygdala of all parkinsonism cases with dementia, often colocalizing with phosphorylated α-syn (pSyn202), though oxidized α-syn (pSyn303) was occasionally observed without colocalization (Forman et al., 2002).

Further evidence emerged from genetic PD cases. Kotzbauer et al. (2004) analyzed the brain of a Contursi family member carrying the A53T α-syn mutation and identified phosphorylated tau in the amygdala and other regions, sometimes colocalized with phosphorylated or nitrated α-syn. In sporadic PD and PDD, hyperphosphorylated tau was detected at Ser262 and Ser396/404 in the striatum of both groups, with Ser202 phosphorylation exclusive to PD. No tauopathy was observed in the inferior frontal gyrus, correlating with a lack of increased p-GSK-3β levels in that region (Wills et al., 2010). Compta et al. found tau pathology in the entorhinal and fusiform cortices of PD and PDD brains, with higher levels in PDD linked to β-amyloid and α-syn, as well as tau presence in the hippocampus and lateral temporal neocortex (Compta et al., 2011).

Studies in neural grafts further supported tau’s involvement in PD. Neural grafts in two PD patients developed hyperphosphorylated tau inclusions and neurofibrillary tangles, with abundant pathology in the cortex and striatum and lesser involvement in the insular cortex, putamen, and caudate (Cisbani et al., 2017). Phosphorylated tau was also localized in neurons, glia, and astrocytes across brain regions of sporadic PD brains, with varying distribution patterns (Moszczynski et al., 2017). Hadi et al. identified three phosphorylated tau forms (cis p-tau, AT100, AT8) in the substantia nigra, with cis p-tau showing the widest distribution (Hadi et al., 2021). More recently, Tu Haitao et al. detected phosphorylated tau (p-tau-S199/202) in the caudate, putamen, and temporal cortex of PDD and LBD brains (Tu et al., 2022).

Although there is consensus on tau localization in some areas of post-mortem PD brains, significant variability exists across studies. These discrepancies may stem from differences in clinical pre-autopsy diagnoses, inconsistent genetic analyses, or classification of brain samples as sporadic PD despite possible genetic underpinnings. Despite these challenges, the evidence underscores the critical role of phosphorylated tau in PD progression and its interplay with α-syn.

Studying tau, α-syn, and other molecular players in post-mortem PD brains is fundamental for developing accurate disease models. Such models are essential for recapitulating the progression of human PD and, ultimately, designing effective therapeutic strategies.

### 3.5 Evidence of misfolded tau in Lewy body dementia

PD is not the only synucleinopathy in which misfolded tau has been detected. In LBD, the aggregation of α-syn often co-occurs with amyloidogenic proteins such as β-amyloid and tau (Table 3). This challenges the notion of isolated proteinopathies, instead highlighting potential interactions between these pathological proteins.

**Table 3:** Tau in Lewy body dementia.

Studied structures	Antibodies	Pathological findings	References
Midbrain	Tau 8,073 [tau tangles] and 8D8 tau [pPHF-*τ* neurofilament]	Microscopic tangles of hyperphosphorylated tau in the cortex. The number of tangles corresponds to the amount of tau	Strong et al. (1995)
Cerebrospinal Fluid	Tau-total and tau-pThr181	Levels of total tau protein and p-tau 181 protein were significantly higher in the DLB and AD groups compared to the non-dement control group	Mollenhauer et al. (2006)
Temporal Cortex	AT8 [pSer202/Thr205]	More significant tau pathology (AT8 +) compared to the control group, but lower than in AD	Arezoumandan et al. (2024)

AD, Alzheimer’s disease; AT8, anti-phospho-tau; LBD, Lewy body Dementia; p, phosphorylated; PHF, paired helical filaments.

In an early study, Strong et al. (1995) observed that abnormally hyperphosphorylated tau was generally absent in LBD, appearing only in cases with detectable neocortical tangles (Strong et al., 1995). Their analysis of 48 subjects—including controls, PD, AD, and LBD cases—revealed that only one LBD case had hyperphosphorylated tau levels comparable to AD, with tangles present in the cortex. Three other LBD cases exhibited some hyperphosphorylated tau with occasional cortical tangles. These findings suggested that hyperphosphorylated tau does not significantly contribute to LBD pathology or cognitive deficits, contrasting its role in AD.

Subsequent studies, however, provided differing evidence. An AD-like cerebrospinal fluid (CSF) profile, characterized by decreased β-amyloid (1-42) and increased total tau or phosphorylated tau (p-tau 181), was identified in a subset of LBD patients and associated with faster cognitive decline. These findings underscore the potential diagnostic value of tau as a biomarker for distinguishing LBD from other neurodegenerative diseases (Di Censo et al., 2020).

Mollenhauer et al. (2006) analyzed CSF levels of β-amyloid (1-40), β-amyloid (1-42), total tau, and p-tau 181 in LBD, AD, and control groups. While total tau and p-tau 181 levels were higher in LBD than controls, they remained lower than in AD. The overlap of tau levels between LBD and AD limited the utility of these markers for definitive differentiation. Additionally, although advanced LBD stages were correlated with increased tau levels, more specific biomarkers are needed for accurate diagnostic discrimination (Mollenhauer et al., 2006).

Di Censo et al. (2020) explored the relationship between AD CSF biomarkers and LBD clinical features in a more extensive cohort study. Their findings linked tau pathology to atypical LBD presentations, characterized by fewer core features, such as parkinsonism, REM sleep behavior disorder, and visual hallucinations. This overlap between LBD and AD pathology resulted in less characteristic clinical profiles, reflecting the complex molecular interactions between α-syn and tau (Di Censo et al., 2020).

More recently, a cohort study investigated the interrelationships between α-syn, β-amyloid, and tau in individuals with autopsy-confirmed neurodegenerative diseases, including LBD. Early tau epitopes correlated positively with α-syn in synucleinopathies, suggesting an interaction between these proteins during disease progression. Conversely, tauC3, a marker of mature tangles, was exclusively associated with β-amyloid and showed no relationship with α-syn. Interestingly, tauC3 negatively correlated with all significant clinical features of LBD, suggesting its role may differ depending on the co-occurrence of β-amyloid or α-syn (Arezoumandan et al., 2024).

These findings collectively demonstrate that misfolded tau plays a complex and variable role in LBD, influenced by its interaction with other pathological proteins. While hyperphosphorylated tau may not be a primary driver of LBD pathology, its association with overlapping AD pathology and atypical clinical presentations highlights the need for further investigation into the molecular and clinical interplay between tau and α-syn. Understanding these interactions will be critical for developing specific biomarkers and therapeutic strategies tailored to the unique features of LBD.

### 3.6 Evidence of misfolded tau in multiple system atrophy

Although tau pathology has been more extensively studied in LBs, evidence of tau involvement in GCIs within MSA has also been reported. While tau’s role in MSA is less pronounced than α-syn, several studies have investigated its presence and distribution in MSA brain regions.

Jellinger et al. examined the morphological differences between MSA with predominant parkinsonism (MSA-P) and MSA with predominant cerebellar ataxia (MSA-C) in patients with cognitive impairment (Jellinger, 2023). They found tau pathology was slightly more pronounced in the MSA-P group, particularly in the cortex and brainstem, in cases with mild to moderate cognitive impairment. These findings suggest that tau may contribute to the cognitive symptoms observed in MSA, although its role appears secondary to α-syn.

A pathological variant of MSA, characterized by severe neuronal cytoplasmic inclusions (NCIs), was described by Ando et al. This variant involved hippocampal granule cells, cornu Ammonis areas, the parahippocampal gyrus, and the amygdala (Ando et al., 2022). Interestingly, minimal levels of hyperphosphorylated tau and phosphorylated TDP-43 aggregates were observed in these cases, reinforcing the idea that α-syn drives MSA pathogenesis more than tau.

Rong et al. conducted a comparative study of α-syn and phosphorylated tau (p-tau) protein levels in PD, MSA, and progressive supranuclear palsy (PSP) (Rong et al., 2021). Their findings revealed the presence of p-tau aggregates in the sural nerves of MSA patients but not in PD patients or healthy controls, suggesting a peripheral component of tau pathology in MSA. This observation introduces the potential utility of analyzing p-α-syn and p-tau levels in sural nerves as biomarkers for distinguishing PD, MSA, and PSP.

These studies collectively indicate that while tau pathology is not a primary driver of MSA, its presence in specific brain regions and peripheral tissues may contribute to the disease’s clinical features and serve as a complementary biomarker for differential diagnosis (Table 4). Further research must elucidate tau’s precise role in MSA and its potential interactions with α-syn in this disease context.

**Table 4:** Tau in multiple system atrophy.

Studied structures	Antibodies	Pathological findings	References
Cortex and Brainstem	AT8 [pSer202/Thr205] (Same for all studies)	Most tau-positive glia was detected in frontal or temporal white matter or putamen.	Jellinger (2023)
Hippocampus		Concomitant LBs were observed in the substantia nigra, locus coeruleus, and dorsal nucleus of the vagus. Deposits of hyperphosphorylated tau and beta-amyloid were generally mild in the hippocampus and limbic areas of the hippocampal MSA patients. Four hippocampal MSA patients showed a few granular TDP-43 aggregations in the hippocampus. Concomitant LB pathology, Braak’s NFT stages, Thal’s amyloid phases, and Saito’s argyrophilic grain stages did not differ between the hippocampal MSA and classical MSA groups.	Ando et al. (2022)
Sural Nerves		P-tau was expressed only in Schwann cells but not in the axon of the sural nerves of MSA. The MSA group colocalised P-tau accurately with S100 β (myelin marker). It was observed that approximately 100% of the p-tau immunoreactivity was positive in sural nerves in MSA patients.	Rong et al. (2021)

AT8, anti-phospho-tau; LB, Lewy body; MSA, multiple system atrophy; NFT, neurofibrillary tangles; p, phosphorylated; TDP-43, Transactive response DNA binding protein of 43 kDa.

## 4 Synergistic effects of interactions between tau and α-synuclein

**Figure 4:**
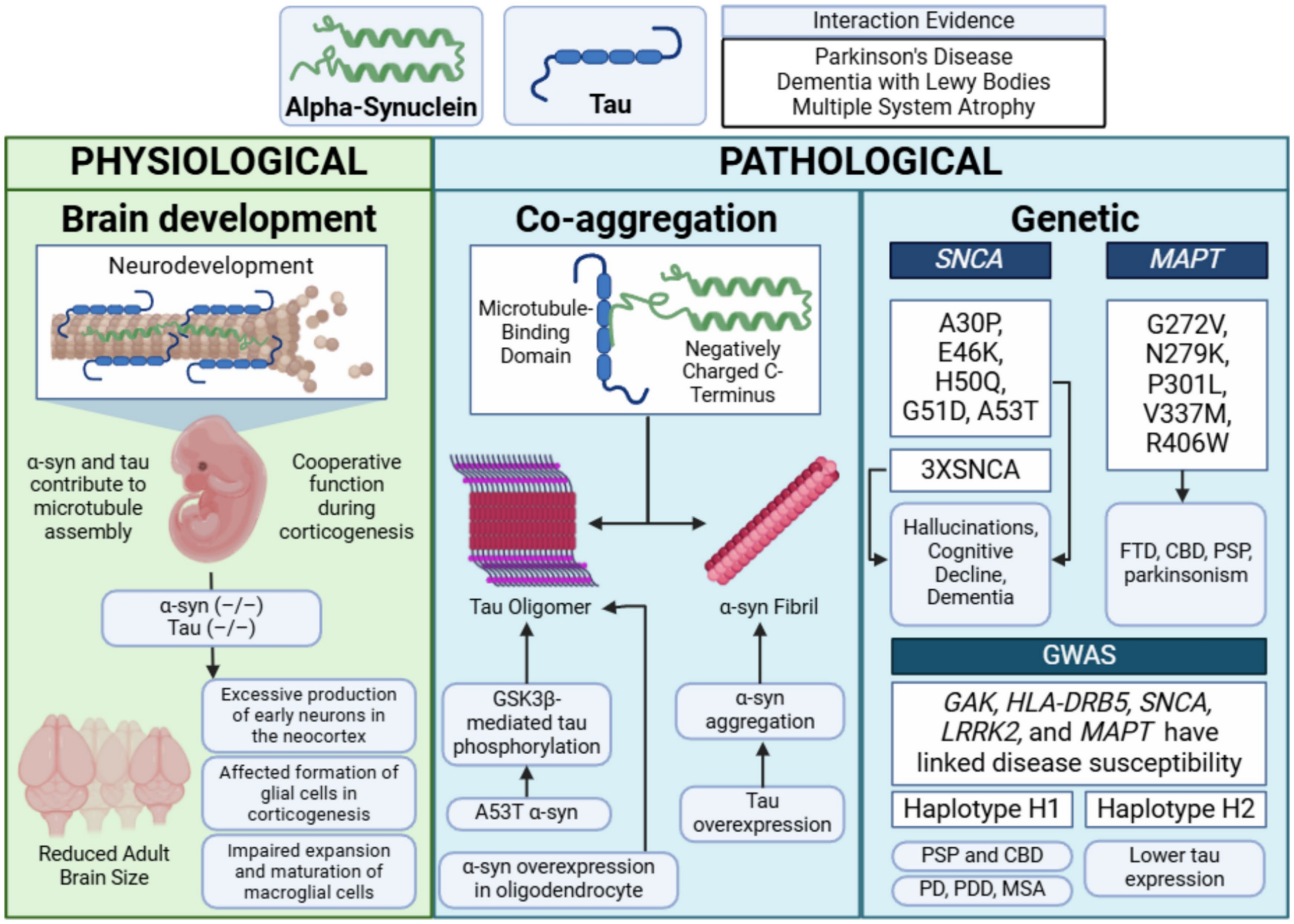
Main synergistic interactions between α-synuclein and tau. Physiological cooperation between tau and α-synuclein has been observed during neurodevelopment, particularly in corticogenesis. Pathological interactions have been demonstrated in *in vitro* models, where the microtubule-binding domain of tau and the negatively charged C-terminus of α-synuclein promote co-aggregation. These interactions are influenced by genetic factors, such as *SNCA* triplications (3X*SNCA*) and *MAPT* mutations. Diseases associated with these mechanisms include Parkinson’s disease (PD), Parkinson’s disease dementia (PDD), multiple system atrophy (MSA), corticobasal degeneration (CBD), and progressive supranuclear palsy (PSP)—figure created with BioRender.com.

The presence of tauopathy in synucleinopathies such as PD and LBD suggests potential mechanisms of synergy between tau and α-syn in both physiological and pathological contexts. Emerging evidence highlights an intricate interplay between these two proteins, with recent research uncovering specific mechanisms by which their interactions may contribute to neurodegeneration (Figure 4).

### 4.1 Physiological cooperation: brain development

While α-syn and tau are primarily studied for their roles in neurodegenerative diseases, their physiological functions remain incompletely understood. Surprisingly, mice lacking α-syn or tau exhibit no overt abnormalities, suggesting potential functional redundancy among neuronal microtubule-binding proteins (Harada et al., 1994; Ke et al., 2012). Both proteins contribute to microtubule assembly and stabilization *in vitro* (Cleveland et al., 1977b; Toba et al., 2017), underscoring their significance in maintaining neuronal structure and function (Harada et al., 1994; Qiang et al., 2018). This section summarizes their physiological cooperation, with a detailed analysis by Jin et al. (2024).

Neuronal microtubules are essential for maintaining cell shape, supporting neurodevelopment, and facilitating axonal transport (Sleigh et al., 2019). As previously described, the physiological interactions between tau and these microtubules are essential for stabilizing the labile domains and assembly. However, α-syn’s implications in such mechanisms have just begun to be considered. The first suggestion of a potential direct interaction between α-syn and tubulin/microtubules in neurons was made in 2001 (Payton et al., 2001). Further experimental evidence from mouse and rat nervous systems (Toba et al., 2017; Amadeo et al., 2021) and post-mortem human brains (Amadeo et al., 2021) have been found. Based on the available literature, various research groups have identified different regions of α-syn as being involved in its interaction with tubulin/microtubules, including primarily the N-terminal region (Cartelli et al., 2016), the NAC region (Zhou et al., 2010), or the C-terminal region (Alim et al., 2004). The significant variations in the proposed interaction sites suggest that the entire α-synuclein sequence may play a role in these interactions. However, further research is necessary to determine the residues responsible for the direct interaction between α-syn and tubulin/microtubules.

Nonetheless, independently of the specific mechanisms regulating physiology, it has been evidenced that mutations in tubulins or microtubule-binding proteins have been implicated in severe neurodevelopmental (Fallet-Bianco et al., 2008; Jin et al., 2017) and neurodegenerative disorders (Calogero et al., 2019; Shafiq et al., 2021). Disruption of α-syn and tau interactions can impair tau-tubulin binding and promote tau hyperphosphorylation, leading to microtubule destabilization and aggregation (Jensen et al., 1999; Moussaud et al., 2014).

Recent findings by Wang et al. demonstrated that mice lacking both α-syn and tau (α-syn–/–tau–/–) exhibit significant neurodevelopmental alterations (Wang S. et al., 2022). While these double-knockout mice showed enlarged brains during embryonic development, their adult brain size was reduced compared to controls. This phenotype was linked to decreased Notch signaling, which accelerated neurogenesis during the early embryonic phase—in utero studies from embryonic day 12 (E12) to E14 revealed increased interkinetic nuclear migration within the ventricular zone and excessive production of early neurons in the neocortex. The overproduction of neurons depleted neural progenitor cells prematurely, leaving fewer progenitors available at mid-gestation. This depletion subsequently affected the formation of glial cells, including oligodendrocytes and astrocytes, during the later stages of corticogenesis.

Additionally, the deletion of α-syn and tau impaired the expansion and maturation of microglial cells, resulting in increased neuronal cell density, reduced brain size, and decreased cortical thickness in postnatal brains compared to controls. These findings emphasize the cooperative roles of α-syn and tau in corticogenesis and highlight their importance in regulating neurogenesis and glial cell formation (Wang S. et al., 2022).

Future research should elucidate how α-syn and tau modulate key pathways, such as Notch signaling, and how their interactions influence microtubule dynamics during neurodevelopment. Understanding these mechanisms could provide valuable insights into the dual roles of these proteins in physiology and pathology.

### 4.2 Neuropathological co-aggregation and cross-seeding theory

Both α-syn and tau are prone to misfolding into pathological fibrils and aggregates and increasing evidence suggests that these proteins interact synergistically in both physiological and pathological contexts. The negatively charged C-terminus of α-syn interacts with tau’s microtubule-binding domain, enhancing each other’s aggregation potential by destabilizing proteostasis (Jensen et al., 1999; Giasson et al., 2003; Nonaka et al., 2010; Lu et al., 2020; Yan et al., 2020). Their fibrillization follows a nucleation-dependent process, with seed-dependent propagation of aggregates (Guo and Lee, 2014). Cross-seeding has been demonstrated in animal models, where the introduction of pathological α-syn or tau into healthy tissue induces aggregation of the other protein (Kordower et al., 2008; He et al., 2020). For example, injecting tau strains from tauopathy brains into 6htau mice (expressing equal ratios of 3R and 4R human tau isoforms) resulted in cell-type-specific tau pathology reflecting the injected isoform (Li et al., 2008).

Prion-like spread of α-syn and tau has been confirmed in clinical contexts. In PD patients who received fetal midbrain neuron grafts, LB-like inclusions were identified in the transplanted neurons years later, indicating host-to-graft propagation of pathological aggregates (Riedel et al., 2009; Guo et al., 2013). The transition of α-syn and tau from soluble forms to insoluble amyloid-like aggregates is a central feature of neurodegeneration, with mutations in *SNCA* or *MAPT* accelerating this process. Different α-syn strains vary in their ability to seed tau aggregation in neuronal cells and human P301S tau transgenic mice (Singh et al., 2019).

Pathological interactions extend to cellular dysfunction. Overexpression of α-syn in oligodendroglial cells promotes tau aggregation (Singh et al., 2019), while the familial PD mutation A53T α-syn induces mislocalization of tau to postsynaptic spines, leading to postsynaptic dysfunction, hippocampal hyperexcitability, and cognitive impairment in PD models (Teravskis et al., 2018; Irwin et al., 2017). A53T α-syn uniquely triggers GSK3β-mediated tau phosphorylation and calcineurin-dependent loss of AMPA receptors, contributing to tau misplacement and exacerbating dementia in transgenic mice (Badiola et al., 2011; Teravskis et al., 2018). Conversely, tau overexpression enhances α-syn aggregation, increasing aggregate size and toxicity (Pan et al., 2022). Tau-modified α-syn fibrils exhibit a higher seeding capacity, leading to more severe motor and cognitive impairments in mice (Pan et al., 2022). This overall disrupting of proteostasis may describe the intertwined mechanisms of associated proteinopathies.

Interestingly, some elderly individuals show Lewy-related and AD-related pathologies at autopsy despite remaining free from parkinsonism or dementia (Knopman et al., 2003; Markesbery et al., 2009; Kok et al., 2022). Approximately 24% of cognitively normal controls exhibit Lewy pathology, while 20–40% display AD-related changes (Knopman et al., 2003; Markesbery et al., 2009). This resistance may reflect either preclinical disease phases or protective mechanisms. For example, mutations in *PRKN* or *LRRK2* lead to neuronal degeneration without α-syn or tau inclusions (Gaig et al., 2007; Calogero et al., 2019). Furthermore, autophagy impairments are strongly linked to neurodegeneration, suggesting that protein aggregates may be protected by sequestering toxic oligomers (Markesbery et al., 2009; Kok et al., 2022).

Combined evidence implicates soluble oligomeric forms of α-syn and tau, rather than their aggregated fibrillar states, as primary drivers of neurodegeneration (Bengoa-Vergniory et al., 2017). These oligomers disrupt cellular homeostasis, synaptic function, and neuronal survival, underscoring the importance of targeting early stages of misfolding in therapeutic strategies.

### 4.3 Genetic interactions

Potential genetic interactions between tau and α-syn may contribute to the overlapping pathologies observed in synucleinopathies and tauopathies. Familial mutations in the *MAPT* or *SNCA* genes often result in clinical presentations that include both parkinsonism and dementia. For instance, familial parkinsonism associated with pathogenic α-syn mutations (e.g., A30P, E46K, H50Q, G51D, A53T) or duplication/triplication of the wild-type *SNCA* gene frequently manifests with atypical features, such as hallucinations, cognitive decline, and dementia (Halliday and McCann, 2010; Nalls et al., 2011; De Marchi et al., 2014; Aarsland, 2016; Fagan and Pihlstrøm, 2017; Kusters et al., 2020; Piredda et al., 2020; Planas-Ballvé and Vilas, 2021).

Mutations in *MAPT* are also implicated in a spectrum of neurodegenerative disorders, including parkinsonism. Early studies linked *MAPT* splice-site and missense mutations (e.g., G272V, N279K, P301L, V337M, R406W) to frontotemporal dementia with parkinsonism-17 (FTDP-17) (Dumanchin et al., 1998; Poorkaj et al., 1998; D’Souza et al., 1999; von Bergen et al., 2001). Subsequent research expanded this list to include numerous intronic and exonic mutations (Clark et al., 1998; Hutton et al., 1998; Spillantini et al., 1998; D’Souza et al., 1999). Specific mutations like P301L and N279K are primarily associated with familial frontotemporal dementia (FTD) (Wszolek et al., 1992; Hutton et al., 1998), while others, such as S305N and K369I, are linked to L-DOPA-responsive parkinsonism (Neumann et al., 2001) and corticobasal degeneration (CBD) (Kouri et al., 2014; Cullinane et al., 2023). Interestingly, mutations like deltaN296 have been associated with familial atypical progressive supranuclear palsy (PSP) (Debnath et al., 2022). Within families, *MAPT* mutations often produce a range of symptoms and onset ages, reflecting the gene’s ability to trigger multiple neurodegenerative pathways. Notably, tauopathy in FTDP-17 patients occurs without LBs, suggesting that tau alone can drive parkinsonism (Lee V. M.-Y. et al., 2004; Jellinger, 2009).

Genome-wide association studies (GWAS) have identified at least 24 genetic loci associated with PD risk, including *SNCA*, *MAPT*, *GAK*, *HLA-DRB5*, and *LRRK2* (Pankratz et al., 2009; Satake et al., 2009; Simón-Sánchez et al., 2009; Hamza et al., 2010; Saad et al., 2011; Spencer et al., 2011). Variations in the *MAPT* gene are classified into two significant haplogroups, H1 and H2, resulting from a ~ 900 kb chromosome inversion. The H2 haplotype, associated with lower tau expression, appears protective against neurodegeneration, while the H1 haplotype increases risk for tauopathies (e.g., PSP, CBD) (Heckman et al., 2019; Valentino et al., 2020) and synucleinopathies (e.g., PD, PDD, MSA) (Zabetian et al., 2007; Setó-Salvia et al., 2011; Labbé et al., 2016). Despite these associations, the specific variants driving disorder risk remain undefined.

In LBD, genetic interactions are less clearly established. A recent GWAS did not find a direct association between the *MAPT* locus and disease susceptibility (Bras et al., 2014). However, smaller studies reported correlations between the H1 haplotype and the severity of brainstem synuclein pathology (Colom-Cadena et al., 2013). Additionally, the *SNCA* SNP rs2572324 has been linked to the extent of neocortical LB and neurofibrillary pathology (Peuralinna et al., 2008), suggesting that tau and α-syn may influence each other’s aggregation, impacting dementia and parkinsonism development.

Additional genetic studies highlight indirect connections between tau and α-syn. For example, an SNP in the RIT2 gene, identified through GWAS meta-analyses, interacts with proteins binding both tau and α-syn (Labbe and Ross, 2014). Increased PD risk has also been linked to SNPs in *GSK3*β, a tau kinase (Kwok et al., 2005). While some studies suggest an epistatic interaction between *SNCA* and *MAPT* variants, with combined risk alleles increasing dementia susceptibility in PD patients (Goris et al., 2007), others report no significant interaction between *SNCA*, *MAPT*, or *LRRK2* polymorphisms (Biernacka et al., 2011).

These findings suggest that genetic variations in tau and α-syn genes can influence disease onset and progression independently or through synergistic interactions. Future research exploring the molecular and genetic interplay between *SNCA* and *MAPT* could uncover novel pathways driving neurodegenerative diseases and identify potential therapeutic targets.

## 5 Toward a new understanding of proteinopathies

### 5.1 Are common therapies possible?

The classification of neurodegenerative diseases as proteinopathies, driven by pathological proteins like α-syn and tau, offers new avenues for disease classification, patient stratifications and potential therapeutic research. Research has demonstrated that interactions between α-syn and tau promote fibrillization and cross-seeding, processes central to disease progression (Jensen et al., 1999). Transgenic mouse models with wild-type or familial PD-associated *SNCA* mutations exhibit tau hyperphosphorylation and misfolding patterns similar to neurofibrillary tangles (NFTs) in human brains (Jensen et al., 1999). Targeting these pathological interactions could inhibit fibrillization and cross-seeding, potentially mitigating disease progression. However, α-syn and tau also play essential roles during corticogenesis, where their absence disrupts the balance between neuronal and glial cells and impairs microtubule dynamics (Wang S. et al., 2022). These developmental roles highlight the need to balance therapeutic interventions to preserve their physiological functions.

The prion-like propagation of pathogenic proteins across cells is increasingly recognized as a pivotal mechanism in neurodegenerative disease progression (Goedert, 2015; Goedert et al., 2017). Soluble oligomeric forms of these proteins, rather than insoluble fibrils, appear to be the primary toxic species driving disease onset and progression (Paleologou et al., 2008; Sengupta et al., 2016). Elevated oligomers correlate with disease severity, suggesting that targeting their release or uptake may offer viable therapeutic strategies.

Efforts to clear soluble oligomers from the brain are ongoing. For PD, monoclonal antibodies targeting α-syn have not yielded positive clinical trial results (Castonguay et al., 2021), leaving levodopa as the primary treatment for motor symptoms. In cases resistant to medication, deep brain stimulation (DBS), implemented since the mid-1990s, has provided symptom relief and reduced medication-related side effects (Limousin and Martinez-Torres, 2008; Hacker et al., 2020). However, DBS involves surgical risks, requires expertise, and has contraindications. Alternatively, magnetic resonance-guided focused ultrasound (MRgFUS), using devices like the FDA-approved Exablate Neuro, offers a non-invasive treatment for PD motor symptoms (Martinez-Fernandez et al., 2020; Wang X. et al., 2022).

In AD, immunotherapy has shown more promise despite initial setbacks with drugs like bapineuzumab, solanezumab, and crenezumab (Salloway et al., 2014; Cummings et al., 2018; Honig et al., 2018). In this regard, important progress has been obtained since the launch of immunization therapy for AD in 1999, when active immunization of Aβ-precursor protein (APP) transgenic mice with synthetic polymers of Aβ was shown to reduce cerebral plaque burden dramatically (Schenk et al., 1999). Indeed, apart from the mentioned, other antibodies like gantenerumab, aducanumab, lecanemab, and donanemab have significantly advanced in clinical development (Jucker and Walker, 2023). These antibodies recognize partly different antigenic sites on oligomers of Aβ, except for donanemab that binds to an specific epitope in plaques (Mintun et al., 2021). All of them, differ in their apparent clinical efficacy and ability to lower plaque load. To date, the antibodies that have achieved the highest reduction in Aβ deposition (over 60% after 18 months of treatment)—lecanemab, donanemab, and aducanumab—have demonstrated signs of slowing clinical decline (Budd Haeberlein et al., 2022; Sims et al., 2023; van Dyck et al., 2023). Importantly, the reduction in Aβ burden was associated with lower levels of phosphorylated tau and glial fibrillary acidic protein (GFAP) in cerebrospinal fluid or blood (Mintun et al., 2021; Pontecorvo et al., 2022). However, directly comparing their clinical effectiveness is challenging due to variations in trial parameters, including dosage, treatment schedules, and the characteristics of the patient populations studied. Furthermore, it is important to highlight that clinical benefits associated with these therapies remain limited, which underscores the need to explore tau-based and other non-amyloid based strategies for AD.

In this regard, recent advances include clinical trials with AADvac1, an anti-tau antibody aimed at reducing NFT accumulation and slowing disease progression (Cullen et al., 2024). Other approaches have focused on oligomeric tau rather than NFTs, such as APNmAb005 (Roberts et al., 2020), E2814 (Sigurdsson, 2024), JNJ-63733657 (Chukwu et al., 2018), and Lu AF87908 (Roberts et al., 2020). Although promising, the clinical trails remain on stages I/II, so further research is still needed before they can represent an option for treatment. Similarly, further understanding fibrillization mechanisms in AD is required to better atune immunotherapies depending on the stage of fibrillization, whether it is oligomers or plaques the most crucial targets.

Structural studies have further advanced our understanding of pathogenic proteins. Using electron cryo-microscopy and solid-state nuclear magnetic resonance, researchers have revealed that amyloid fibrils exhibit polymorphic structures. For instance, tau fibrils from AD brains (Fitzpatrick et al., 2017; Falcon et al., 2018) and α-syn filaments from MSA and LBD brains display extensive structural variations (Schweighauser et al., 2020). These polymorphisms suggest that different fibril conformations are associated with distinct neurodegenerative diseases (Fan et al., 2023). Understanding these structural differences may improve diagnostics, improve disease classifications, and once the specific pathogenic forms of the proteins are fully understood, refine therapeutic targets by guiding the development of drugs to inhibit the identified pathogenic pathways responsible for fibril formation.

These insights suggest that common therapeutic strategies targeting shared pathological mechanisms, such as protein aggregation and oligomer formation, could benefit multiple neurodegenerative diseases. However, the complex physiological roles of α-syn and tau necessitate careful consideration of unintended effects, particularly in neurodevelopment and normal cellular function.

## 6 Discussion

The study of synucleinopathies has historically focused on α-syn as the primary pathological driver. However, mounting evidence suggests that LBs and GCIs are not simplistic aggregates of α-syn but complex microenvironments harboring diverse proteins, including ubiquitin, autophagy regulators, and cytoskeletal components (Papp et al., 1989; Murayama et al., 1992; Beyer and Ariza, 2007; Wakabayashi et al., 2007). This diversity points to the multifactorial nature of synucleinopathies, where disruptions in proteostatic networks, rather than a single misfolded protein, drive neurodegeneration. Expanding the scope of research to characterize these inclusions fully could unlock novel therapeutic targets and redefine our understanding of neurodegenerative diseases.

A key element in these inclusions is ubiquitin, a critical regulator of proteostasis. Ubiquitin dysfunction has been implicated in the accumulation of α-syn and the failure to clear co-aggregating proteins, exacerbating cellular stress (Murayama et al., 1992). Similarly, autophagy-related proteins, such as LC3, p62/SQSTM1, and AMBRA1, have been identified in LBs and GCIs, underscoring the role of impaired autophagic and proteasomal pathways in the formation of pathological inclusions (Kuusisto et al., 2001; Olanow et al., 2004; Miki et al., 2018). The presence of cytoskeletal proteins like tau, tubulin, and TPPP/p25 in these inclusions further highlights the destabilization of neuronal and glial structural integrity as a contributing factor to neurodegeneration (Murayama et al., 1992; Kovács et al., 2004; Song et al., 2007). These findings collectively support a unifying hypothesis that synucleinopathies result from the convergence of disrupted proteostatic mechanisms rather than the isolated misfolding of α-synuclein.

One promising direction for therapeutic development involves targeting pathological interactions within these complex aggregates. For instance, the interaction between TPPP/p25 and the unstructured C-terminus of α-syn has been shown to drive α-syn aggregation in a substoichiometric manner (Song et al., 2007). Oláh and Ovádi have proposed a precision-targeted strategy to disrupt this interaction: in PD, TPPP/p25 could be neutralized with an α-syn fragment, while in MSA, α-syn could be targeted using a TPPP/p25 fragment, as these proteins have disease-specific roles in neurons and oligodendrocytes, respectively. This approach exemplifies the potential of disease-specific interventions to minimize off-target effects while preserving the physiological roles of individual proteins.

Achieving such precision requires innovative tools. Computational modeling, molecular docking, and crystallographic studies powered by artificial intelligence could elucidate the molecular determinants of pathological interactions (Kovács et al., 2004; Tanji et al., 2007). Thermodynamic profiling of these interactions would further enhance our understanding of their stability and specificity, paving the way for rational drug design. Additionally, cellular reprogramming approaches aimed at restoring homeostatic mechanisms may offer an alternative strategy to prevent aggregation by maintaining protein balance. However, it is important to highlight that a significant limitation in the current research on seeding and prion-like propagation as mechanisms for the spread of α-synuclein and tau pathology is that these models are highly artificial, raising concerns about their translational relevance to human disease. Most studies rely on transgenic mouse models that grossly overexpress mutant forms of disease-linked proteins, often in a highly selective manner and on a limited number of genetic backgrounds. Such artificial conditions may not accurately reflect the complex interplay of genetic and environmental factors in human neurodegenerative diseases. Developing more physiologically relevant models is essential to understand better the pathogenic mechanisms driving the co-aggregation of α-synuclein and tau.

Recognizing that synucleinopathies are proteostatic collapse diseases demands a paradigm shift in research priorities. A comprehensive examination of the proteomic landscape within LBs and GCIs and the integration of insights from diverse neurodegenerative diseases may reveal shared aggregation pathways and novel therapeutic opportunities. For example, studying the interplay between α-syn and tau has highlighted their potential synergistic roles in driving neurodegeneration through cross-seeding and prion-like propagation (Jensen et al., 1999; Guo and Lee, 2014; Lu et al., 2020). These mechanisms underline the importance of targeting soluble oligomeric forms of these proteins, which are increasingly recognized as the primary drivers of neurotoxicity (Bengoa-Vergniory et al., 2017).

In addition, new research could benefit from a further paradigm shift in the definition of LBs and their role in synucleinopathies. As mentioned above, although these inclusions have traditionally been considered responsible for neurodegeneration in pathologies such as PD and LDB, more and more research suggests the role of α-syn fibrils (and other proteins involved in LBs) in these processes rather than inclusions. In fact, for more than 20 years, authors such as Olanow et al. have proposed that LBs consist of failed aggresomes (Olanow et al., 2004). Aggresomes are cytoprotective proteinaceous inclusions formed at the centrosome that segregate and facilitate the degradation of excess amounts of unwanted and possibly cytotoxic proteins. From this definition, Olanow et al. propose that LBs act by removing potentially cytotoxic proteins but become “permanent garbage cans” instead of functional aggresomes, “acting as recycling plants.” Based on this logic, a focus on research to better understand the impact of more logical culprits such as oligomers or fibrils rather than inclusions (including LBs, GCIs, and neurofibrillary tangles)—despite how well their accumulation correlates with disease—may allow for more accurate and precise approaches to the true cytotoxic factors. Nevertheless, further research is needed to reconcile the apparent disparities and to ensure, once and for all, who is the true culprit in synucleinopathies.

In conclusion, future research must prioritize an inclusive approach considering synucleinopathies’ multifactorial nature. By reframing these disorders as interconnected proteinopathies, we may catalyze the development of innovative, multifaceted treatment strategies. This integrative perspective can potentially redefine neurodegenerative disease research, advancing our ability to address the complex interplay of proteins, pathways, and cellular networks that underpin these devastating conditions.
